# Berry phase and band structure analysis of the Weyl semimetal NbP

**DOI:** 10.1038/srep33859

**Published:** 2016-09-26

**Authors:** Philip Sergelius, Johannes Gooth, Svenja Bäßler, Robert Zierold, Christoph Wiegand, Anna Niemann, Heiko Reith, Chandra Shekhar, Claudia Felser, Binghai Yan, Kornelius Nielsch

**Affiliations:** 1 Institute of Nanostructure and Solid-State Physics, University of Hamburg, 20355 Hamburg, Germany; 2 IBM Research GmbH, 8803 Rueschlikon, Switzerland; 3 Leibniz Institute for Solid State and Materials Research (IFW) Dresden, 01171 Dresden, Germany; 4 Max Planck Institute for Chemical Physics of Solids, 01187 Dresden, Germany; 5 Max Planck Institute for the Physics of Complex Systems, 01187 Dresden, Germany

## Abstract

Weyl semimetals are often considered the 3D-analogon of graphene or topological insulators. The evaluation of quantum oscillations in these systems remains challenging because there are often multiple conduction bands. We observe de Haas-van Alphen oscillations with several frequencies in a single crystal of the Weyl semimetal niobium phosphide. For each fundamental crystal axis, we can fit the raw data to a superposition of sinusoidal functions, which enables us to calculate the characteristic parameters of all individual bulk conduction bands using Fourier transform with an analysis of the temperature and magnetic field-dependent oscillation amplitude decay. Our experimental results indicate that the band structure consists of Dirac bands with low cyclotron mass, a non-trivial Berry phase and parabolic bands with a higher effective mass and trivial Berry phase.

Topological insulators, Dirac semimetals and most recently Weyl semimetals (WSM) are the subject of considerable research interest in both fundamental physics^1^,^2^,^3^ and with respect to applications^4^,^5^,^6^. The band structure of a WSM exhibits a crossing of two bulk bands, which results in two so-called Weyl points with opposing parity^7^,^8^. These pairs are expected to be notably robust but are only realised in 3D systems, where either time-reversal or inversion symmetry is broken^3^,^7^,^9^. Similar to the well-known 2D case in graphene or topological insulators, there is no energy gap, and a linear dispersion relation is present in all directions in k-space away from a single Weyl point^3^,^10^. On the surface of a Weyl metal, Fermi arcs were theoretically predicted^3^ and experimentally shown *via* angle-resolved photoelectron emission spectroscopy (ARPES) for NbP^11^ and other Weyl metals^12^,^13^,^14^,^15^. Both Weyl fermions and surface states are expected to cause numerous exotic quantum effects^2^. Low effective masses are expected for Dirac systems, and with the high mobilities, extremely large magnetoresistance (MR) effects have been observed, particularly in NbP with up to 8 · 10^5^% MR at cryogenic temperatures and 250% at room temperature and 9 T^5^. Particularly interesting for high-performance electronics are their expected and recently demonstrated ultrahigh mobilities^5^,^7^. These characteristic traits of the materials may become important for future device applications such as magnetic field sensors or transistors^16^.

Moreover, Weyl and Dirac semimetals exhibit new and exotic quantum effects such as chiral anomaly and negative magnetoresistance because of their non-trivial topology and associated Berry Phase. However, in contrast to other Weyl metals such as TaAs, the spin orbit coupling in NbP is much weaker due to the lower atomic mass of Nb, which may lead to the existence of additional, parabolic semimetal bands apart from the Dirac bands^7^. Because ARPES measurements resolve the surface states, which only allow for indirect investigation of the bulk band structure, the quantum oscillations must be analysed to reconstruct the Fermi surface. Electric measurements on NbP single crystals have shown strong Shubnikov-de Haas (SdH) oscillations and evidence for Dirac-like dispersions^5^,^7^, but the individual conduction band’s Berry phases have not been clearly analysed. In fact, *ab-initio* band structure calculations have predicted that the conduction bands in NbP will generally be trivial^17^ because the theoretical position of the Fermi level encompasses the Weyl nodes. To realize a Berry phase, the chiral anomaly and other related effects to Weyl metals, the Fermi level must be as close as possible to the Weyl points. A possible route may be controlled electron doping, which will shift the Fermi level to a more desired position, but in reality, due to slight variations during material synthesis, an uncontrolled shift on the order of a few meV may easily be induced.

In this publication, we present de Haas-van Alphen (dHvA) measurements of a NbP single crystal with an intrinsic Fermi level as close as 3.7 meV to the Weyl nodes. A 9 T physical property measurement system (*Quantum Design*) was used with a mounted option to enable Vibrating Sample Magnetometry (VSM). For band structure investigations, VSM is more beneficial than electric measurements because of the trivial sample mounting and the possibility of easily rotating the sample to investigate different crystal orientations while maintaining the possibility to extract transport properties such as effective mass and carrier mobility. Along the major crystal axes ([100] or *k*_*x*_, [010] or *k*_*y*_ and [001] or *k*_*z*_) of the body-centred tetragonal NbP crystal^5^,^18^, we find two fundamentally different behaviours, which are associated to the coexistence of Dirac and parabolic bands. We fit the experimental data to a superposition of up to seven sinusoidal, independent functions, each of which has a frequency, a phase and a damping parameter. The fit enables us to independently extract all relevant parameters. We observe that two bands exhibit relatively low cyclotron masses and a non-trivial Berry phase, and we find evidence for parabolic bands with comparably higher effective masses and trivial Berry phases.

## Results and Discussion

**Figure 1:**
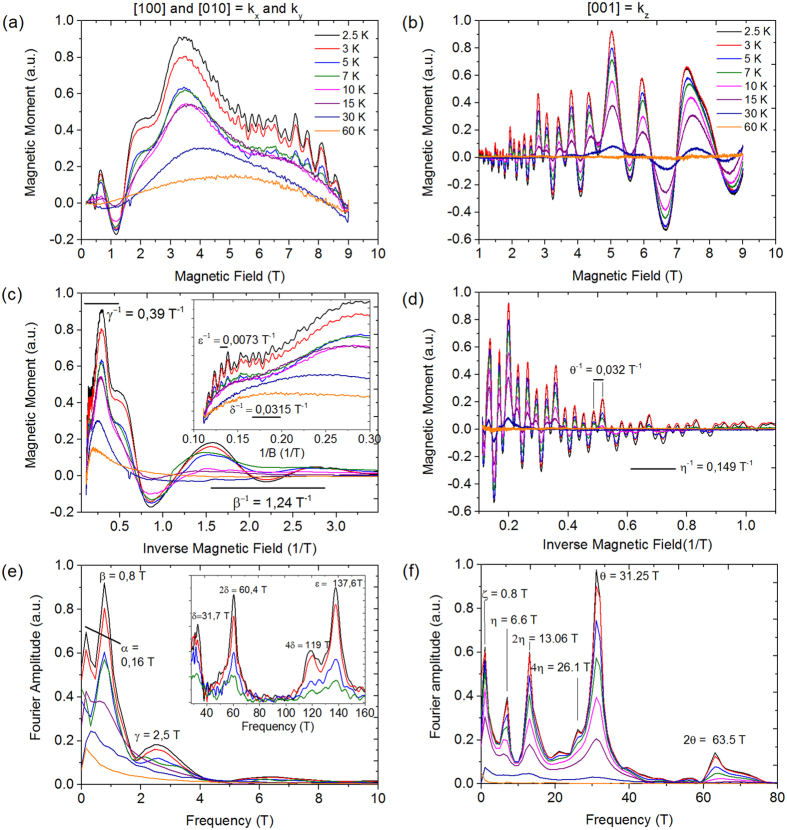
Left column shows the [100] and [010] orientations (*k*_*x*_ and *k*_*y*_, respectively). The right column shows [001] or *k*_*z*_. (**a,b)** Display dHvA oscillations of the magnetic moment as a function of the magnetic field between 2.5 K and 60 K. In the graphs, the raw data are shown with a subtracted linear background. Several superpositions of different oscillation frequencies are visible. (**c,d)** Display the identical data in 1/B. The inset in (**c**) displays the narrow region that corresponds to large magnetic fields >3 T. (**e,f)** Show the fast Fourier transforms of the measurement data. Several oscillation peaks and their higher harmonics can be observed. The small peak at 21 T is neglected in the data evaluation because it may be noise or a notably weakly contributing band. In any case, a temperature dependence of the peak height cannot be extracted. (**f**) The inset in (**e**) displays the higher frequency oscillations.

The experimental data in Fig. 1 show the superpositions of multiple quantum oscillations of the magnetic moment as a function of the applied (a,b) and inverse magnetic field (c,d) and the respective Fourier transforms (e,f) for all three crystal orientations.

Fourier analysis is simultaneously conducted over the entire inverse magnetic field range. For the [100] and [010] directions, the transformation yields several peaks that were indexed by *α*, *β*, *γ*, *δ*, *ε* and higher harmonics. For [001], we find three peaks indexed by *ζ*, *η* and *θ*. We attribute peaks *α* and *ζ* to a general background curvature of the measurement data with no physical relevance. All other bands can be identified as different bulk conduction bands. For *δ* and *η*, we assign higher-order harmonic numbers to peaks where the frequency is harmonically related; however, the higher harmonic has a larger intensity (Fig. 1e,f), which is contrary to common expectation. A possible reason for this effect is the spin orbit coupling, which induces spin splitting, as discussed by Jalan *et al*.^19^ and Moetakef *et al*.^20^. Additionally, the higher harmonics may be the result of non-sinusoidal components in the oscillations, which can easily occur due to noise.

**Figure 2:**
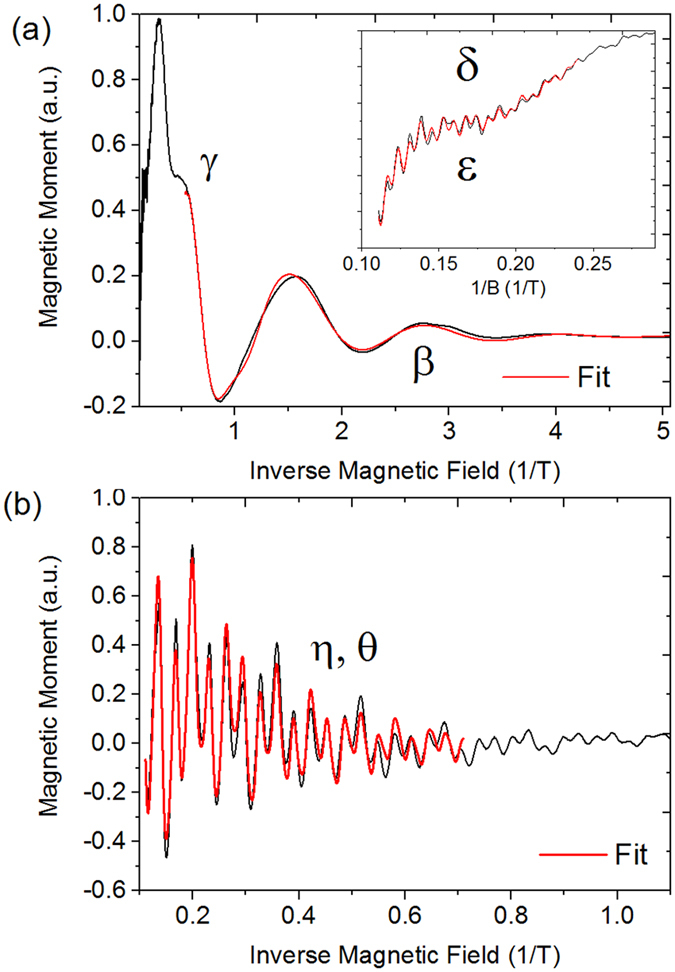
Fits of Eq. 1 to the raw data at 2.5 K for (**a)** *k*_*x*_ and *k*_*y*_ and (**b)** *k*_*z*_. The full formula including all frequencies is always fitted; however, the fitting interval in 1/B is adjusted to a smaller region as shown in the graphs.

The raw data (Fig. 1a,b) show multiple oscillations with different frequencies and magnitudes. If only one type of oscillation is present, it is straightforward to calculate the Landau level diagrams, temperature and field-dependent damping of the amplitudes. However, in our case, only the most prominent oscillations can be distinguished by eye, and determining any damping from the raw data is elusive. Therefore, following the argumentation of Tian *et al*.^21^ and Hu *et al*.^22^, we fit the raw data for T = 2.5 K with a superposition of damped sine-functions for each frequency as in Eq. (1)
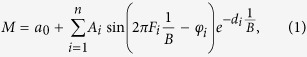
where we sum over the frequencies (*F*_*i*_ with *i* = *α*, *β*, *γ* …) from the Fourier transform. The fit parameters are the associated oscillation amplitude *A*_*i*_, damping factor *d*_*i*_, phase *φ*_*i*_ and a global offset *a*_0_. If higher harmonics are visible, they are incorporated into the sum, but they share an identical damping factor and phase to the respective first harmonic. Note that for oscillations in the magnetic moment, a sine function must be used, whereas the oscillations in the resistivity oscillate as a cosine function^23^, which is important in the Berry phase calculation. Figure 2 shows that Eq. 1 fits the experimental data well, which enables us to extract the individual properties of each band using Onsager relations and the Lifshitz-Kosevich-Shoenberg (LKS) formula^23^. Note that we cannot fit the *k*_*x*_ and *k*_*y*_ data between 0.25 and 0.5 T^−1^ because the *β*-band reaches its quantum limit and stops oscillating as early as 3 T. In addition, the SdH oscillations become visible as early as 0.2 T, which corresponds to a magnetic length of 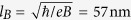 and emphasizes the high sample quality^17^.

If a sine function is used as in Eq. 1, the phase is 
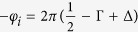
. 2*π*Γ is the Berry phase, and Δ is an additional phase shift between ±1/8, which may arise because of the corrugated Fermi surfaces^24^,^25^. Note that if the dHvA oscillation minima are manually evaluated, an additional summand of 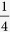 is necessary^26^. In this case, Lifshitz-Onsager quantization rule is 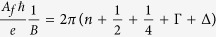.

We emphasize that the precise determination of the Berry phase is often a challenging procedure because the axis intercept is inherently prone to scattering, particularly if only high-index Landau levels can be resolved. However, we can fit the raw data over a multitude of oscillation periods while considering all other superposed oscillations. Thus, we can extract the axis intercept with high accuracy. The standard error from the fits to the phase is less than 1% in all cases, except the δ−band, which is not evaluated because only three periods can be fitted. To cross check our calculations, all minima that are unambiguously visible in the raw data are also manually counted with identical results. The errors are further assessed as shown in the supporting information.

In the [100] and [010] directions, we identify *β* as a Dirac band with an axis intercept of Γ + Δ = 0.48(1), which displays a non-trivial Berry phase. A similar behaviour is found in the [001] direction, where *θ* shows an axis intercept of Γ + Δ = 0.54(5). All other bands have axis intercepts of Γ + Δ < 0.25, which indicates a parabolic behaviour. We investigate the effective mass to further elucidate the carrier characteristics, band topology and whether the fermi surface has been shifted to a more desired position, as previously introduced.

**Figure 3:**
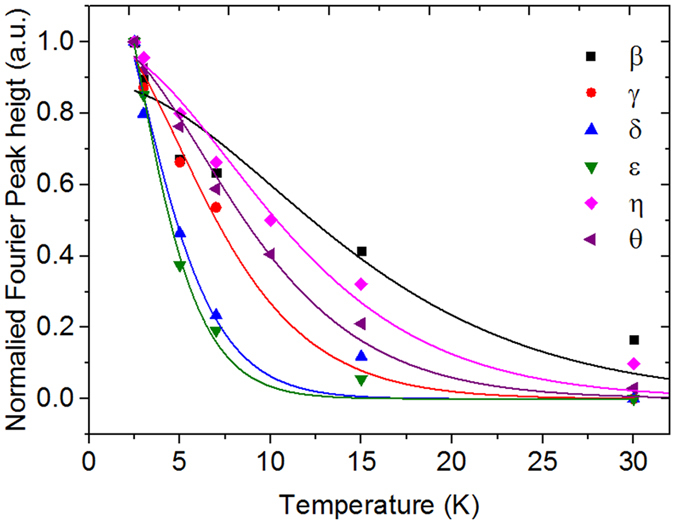
Best fits to the T-dependent LKS plots for all bands. There is a slight mismatch between the points and the fit at higher temperatures due to noise in the FFT data, which can lead to the determination of slightly lower effective masses than those in reality, particularly in *ε* and *θ* (blue and green graphs). Note that the formula only weakly depends on the values for higher temperatures, and the standard errors from the fitting procedure are on the order of 5%. From the quantization condition 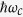 ≥ *k_B_T*, we expect oscillations from Weyl-pocket bands and consequently lower effective masses to sustain at higher T, as shown for the *β* band^7^.

From the Onsager relation *A*_*f*_ = *F* · 2*π*^2^/*ϕ*_0_ with *ϕ*_0_ as the magnetic flux quantum, we determine the size of the Fermi surface cross section; if we approximate it as a circle, the k-vectors are *A*_*f*_ = *πk*^2^. The values are summarized in Table 1. The effective mass is calculated using the temperature-dependent part of the LKS theory, as shown in Eq. (2):
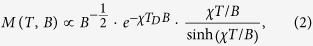
with *χ* = 4*π*^3^*k*_*B*_m*/*eh* as the fitting parameter^5^,^23^,^27^,^28^. *k*_*B*_ is Boltzmann’s constant, *T*_*D*_ is the Dingle temperature, m* is the effective mass and *e* is the elemental charge. A well-pronounced, single oscillation is often selected (imposing *B* = const.), and its amplitude decay with temperature is fitted to Eq. (2). If several oscillations with severely different damping and amplitudes are superposed, this procedure cannot be performed. Therefore, we fit the temperature decay of the peak heights of the Fourier spectra^29^. To calculate m*, *B* must be set to the centre point of the magnetic field interval where the respective oscillations are present, e.g., 5.5 T if the oscillations are visible at 2–9 T^22^. The graphs of the LKS fits are shown in Fig. 3. The respective values of the effective mass are summarized in Table 1. As noted above, the cyclotron mass of the Dirac band, 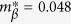, is found to be significantly lower than that for all other bands. We therefore believe that *β* lies within the Weyl pocket. For the *θ*-Band, we find an effective mass of 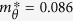, which is of a similar magnitude as the parabolic *γ* or *η* band. These findings may show that deriving conclusions about the existence of Weyl points simply from the Berry phase may not be sufficient, especially because the additional phase shift Δ = ±1/8 allows for additional freedom.

The Dingle temperature 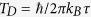 is inversely proportional to the carrier lifetime *τ* and mobility *μ* = *eτ*/m*^23^. For *T* = 2.5 K, the field-dependent damping of the dHvA amplitude (*A*) is known from the fit procedure of the raw *M* vs. 1/*B*-data to Eq. (1).
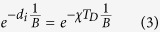
The Dingle temperature, carrier lifetime and mobility are summarized in Table 1. Similar to the effective- mass evaluation, we find a significantly larger mobility for *β* than for all other bands, which further indicates that it may indeed be part of the Weyl pocket. When evaluated individually for each band, the mobility of *β* is two orders of magnitude smaller than reported by Shekhar *et al*. and Wang *et al*. from Hall measurements^5^,^7^. By determining the mobilities from Hall measurements, one imposes a one-carrier and one-channel model, which may explain a significant deviation, particularly because NbP shows a notably large magneto resistance. This magneto resistance results from the electron-hole-resonance, which significantly affects any type of electric transport.

In the parabolic case, the energy distance of the Fermi level is calculated by 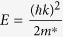 as summarized in Table 1 (Dirac case lacks the factor 1/2). As expected, the Dirac *β*-band lies very close to the Fermi energy (3.74 meV), whereas the energy distance of *θ* is unexpectedly large (42.2 meV). The band structure calculations predict two Weyl pockets: one approximately 5 meV above the Fermi energy and another 57 meV below^17^. The latter does not contribute to the conduction. Considering the Berry phase, low effective mass and close proximity to the Fermi energy, we can safely assign *β* to the first Weyl pocket. Thus, we reinforce the theoretical predictions that a slight shift of the Fermi level towards the Weyl points may incur numerous exotic effects^17^. New experiments on the electric transport in structured NbP thin films are in preparation. The finding of nontriviality for the *θ*-Band raises an open question of whether the analysis of the Berry phase alone can strongly prove the existence of Weyl points.

### Conclusion and Outlook

In summary, we analyse the bulk magnetometry of the Fermi surface of the Weyl semimetal NbP for the fundamental crystal orientations. We demonstrate that a fit of the raw data enables us to separate superposed oscillations. Low effective masses and nontrivial Berry phases are obtained for one band, which experimentally proves that a slight shift of the Fermi level activates the Weyl nodes in the electric transport of NbP. Simultaneously, we find evidence for parabolic bands with higher effective masses and zero Berry phase, which confirms that the low spin-orbit coupling strength enable the presence of both types of bands in NbP. We emphasize that a careful, individual assessment of each conduction band is important to correctly interpret the quantum oscillatory measurement data. Positioning the Fermi level as close as possible to the Weyl nodes is crucial in the ongoing experimental effort to characterize Weyl semimetals. For potential applications of Weyl semimetals in electric devices, a route to miniaturize the bulk crystals must be developed. We propose using tailored growth substrates in a crystal growth furnace or using a focused ion beam to shape the desired nanostructures. The effect of possible surface states will be of great interest, such as in thickness-dependent thin-film studies.

## Methods

Polycrystalline NbP powder was synthesized in a direct reaction of Nb powder (99.9%) and red phosphorous pieces (99.999%) in an evacuated silica tube for 48 h at 800 °C. Single NbP crystals were grown from this powder in a chemical transport reaction with iodine as a transport agent. The source and sink were set to 950 °C and 850 °C, respectively. The VSM measurements were conducted in a *Quantum Design PPMS* cryostat with a VSM Option.

## Additional Information

**How to cite this article**: Sergelius, P. *et al*. Berry phase and band structure analysis of the Weyl semimetal NbP. *Sci. Rep.* **6**, 33859; doi: 10.1038/srep33859 (2016).

## Supplementary Material

Supplementary Information

## Figures and Tables

**Table 1:** *α* to *ε* displays [100] and [010] or *k*_*x*_ and *k*_*y*_, whereas *ζ* to *θ* displays [001] or *k*_*z*_.

	*F* (T)	Phase Γ + Δ	m* (*m*_0_)	*A*_*Fermi*_ (10^−5^ Å^−2^)	*k* (10^−3^ Å^−1^)	*E* (meV)	*τ* (10^−12^ s)	*μ* (cm^2^/Vs)
*α*	0.16							
*β*	0.8	0.48(1)	0.048	7.64	4.93	3.74	0.73	25800
*γ*	2.5	0.19(5)	0.110	23.9	8.72	2.62	0.42	6700
*δ*	31.7	—	0.183	303	31.0	39.7	0.33	3200
*ε*	137.6	0.24(7)	0.255	1314	64.7	62.4	0.30	2100
*ζ*	0.98							
*η*	6.6	0.15(0)	0.086	63.0	14.2	8.93	0.36	7400
*θ*	31.25	0.54(5)	0.086	298	30.8	42.2	0.37	7600

A discussion of the errors can be found in the supporting information. The columns correspond to the oscillation frequency *F*, phase Γ + Δ, effective mass m*, size of the Fermi surface *A*_*Fermi*_, corresponding *k-*vectors, energy distance from the Fermi level *E,* scattering lifetime *τ* and mobility *μ*. Note that Γ + Δ is the value of the phase extracted from the fits to Equation (1) and Δ = ±1/8 has not been added in either direction yet.
