# Supplementary material for: Berry phase and band structure analysis of the Weyl semimetal NbP
Source: Sci Rep. 2016 Sep 26;6:33859. doi: 10.1038/srep33859 (PMC5036179; doi:10.1038/srep33859)
Supplement: Supplementary Information [file srep33859-s1.pdf]

Supporting information for the manuscript *Berry Phase and band structure analysis of the Weyl semimetal NbP*

Philip Sergelius, Johannes Gooth, Svenja Bäßler, Robert Zierold, Christoph Wiegand, Anna Niemann, Heiko Reith, Chandra Shekhar, Claudia Felser, Binghai Yan, Kornelius Nielsch

Multiband fitting procedure

In this chapter of the supporting information, more details on the fitting procedure of the raw data to Eq. 1 is given. We emphasize that the amplitude needs to be fixed to positive values, because a negative amplitude corresponds to a phase shift of π. After a successful fitting, the individual oscillations can be extracted and plotted independently of each other, as can be seen in Figure SI-1 and SI-2. Only the fundamental frequencies are shown, not any higher harmonics. A comparison with the raw data used in Figure 2a reveals a good agreement.

$$M = a_0 + \sum_{i=1}^{n} A_i \sin(2\pi F_i \frac{1}{B} - \varphi_i) e^{-d_i \frac{1}{B}}, \quad (1)$$

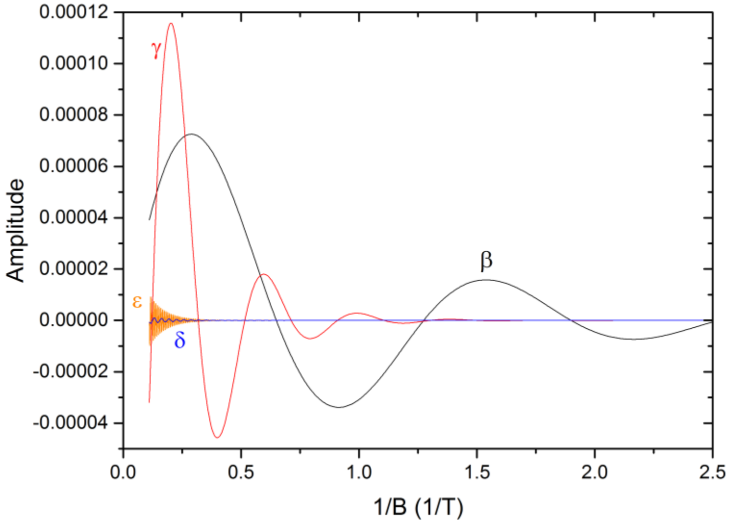

Figure SI-1: From the fits to Eq. 1 the individual oscillations are extracted for each band in the k_x and k_y direction.

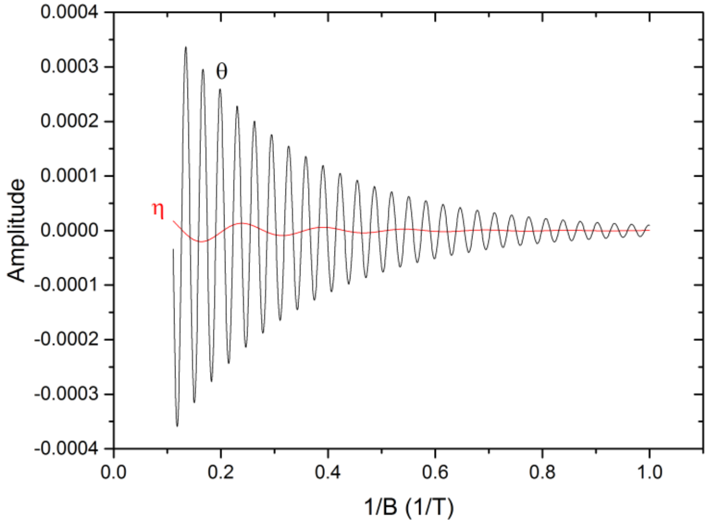

Figure SI-2: From the fits to Eq. 1 the individual oscillations are extracted for each band in the k_z direction.

The fits were conducted in Origin 9.1 and the standard errors of the phase given by the fitting algorithm is less than 1%, except for the δ −band where it is 14%, because the amplitude is comparably small and only three oscillation periods are within the fitting interval. The standard error of the amplitudes A_i is <0.1% for β, 5% for γ, 40% for δ, 20% for ε, 10% for η and 2% for θ. For the damping factors d_i the standard errors are 0.4% for β, 1% for γ, 30% for δ, 12% for ε, 3% for η and 2% for θ

For optical comparison, the minima of the separated oscillation peaks shown in Figure SI-1 and SI-2 can be plotted as landau level graphs as in Figure SI-3. The Berry Phase itself is calculated directly from the phase given by Eq. 1.

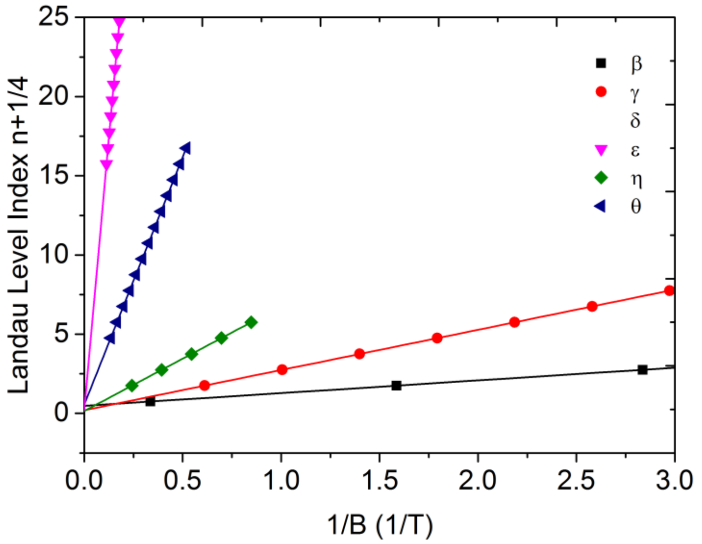

Figure SI-3: Landau level graphs for all bands. The higher the slope, the more prone to error is the axis intercept.
